# Role of specialized pro-resolving mediators on inflammation, cardiometabolic health, disease progression, and quality of life after omega-3 PUFA supplementation and aerobic exercise training in individuals with rheumatoid arthritis: a randomized 16-week, placebo-controlled interventional trial *

**DOI:** 10.12688/f1000research.138392.1

**Published:** 2023-08-07

**Authors:** Sebastián Jannas-Vela, Alejandro A Candia, Luis Peñailillo, Paola Barrios-Troncoso, Jeremy Zapata-Urzúa, Joanny Rey-Puente, Harold M Aukema, David M Mutch, Rodrigo Valenzuela, Denisse Valladares-Ide

**Affiliations:** 1 Instituto de Ciencias de la Salud, Universidad de O'Higgins, Rancagua, O'Higgins Region, Chile; 2 Exercise and Rehabilitation Sciences Institute, Universidad Andres Bello, Santiago, Santiago Metropolitan Region, Chile; 3 Hospital Regional Rancagua Libertador Bernardo O'Higgins, Rancagua, O'Higgins Region, Chile; 4 Department of Food and Human Nutritional Sciences, University of Manitoba, Winnipeg, Manitoba, Canada; 5 Department of Human Health and Nutritional Sciences, University of Guelph, Guelph, Ontario, Canada; 6 Department of Nutrition, Universidad de Chile, Santiago, Santiago Metropolitan Region, Chile

**Keywords:** Omega-3, exercise, rheumatoid arthritis, oxylipins, specialized pro-resolving mediators, inflammation, gene expression.

## Abstract

**Background:** Rheumatoid arthritis (RA) is a chronic inflammatory disease characterized by autoantibody production and synovial membrane damage. It significantly impairs overall function and quality of life. Consumption of omega-3 (n-3) polyunsaturated fatty acids (PUFAs) and regular aerobic exercise (AEx) training are reported to have positive effects on the progression of RA. However, the mechanisms behind these benefits are still inconclusive. This study protocol will investigate the effects of n-3 PUFA supplementation and AEx training on disease progression, cardiometabolic health, and quality of life, and their association with the plasma and synovial fluid levels of specialized pro-resolving mediators (SPMs) in subjects with RA.

**Methods:** The study consists of a 16-week intervention period, during which participants will be randomly assigned in a double-blinded manner to one of four groups: placebo control (PLA), PLA+AEx, n-3, or n-3+AEx. The PLA groups will be given a gelatin-filled capsule, while the n-3 groups will be given n-3 PUFAs equivalent to 2.5 g/d of docosahexaenoic acid and 0.5 g/d of eicosapentaenoic acid. The AEx groups will perform exercise three times per week on a stationary electronically braked cycle ergometer at 60-70% of their VO2peak for 50-60 minutes. Before and after the intervention, participants will undergo RA-specific and functional measurements, peak aerobic capacity test, and a dietary and physical activity assessment. Venous blood and synovial fluid from the knee joint will be collected. Changes in disease progression, cardiometabolic health, and quality of life, as well as erythrocyte membrane composition to assess n-3 incorporation, SPM levels, inflammatory markers, and gene expression from blood and synovial fluid will be analyzed.

**Conclusions:** The study aims to elucidate the SPMs that regulate the inflammatory gene expression pathways and associate them with the improvements in disease progression, cardiometabolic health, and quality of life after n-3 PUFA supplementation and AEx training.

**Registration**: ClinicalTrials.gov #NCT05945693.

## Introduction

Rheumatoid arthritis (RA) is an autoimmune and chronic inflammatory disease characterized by autoantibody production [rheumatoid factor (RF) and anti–citrullinated protein antibody] and damage of the synovial membrane of the joints, causing swelling, pain, stiffness, and fatigue, among other symptoms. ^1^^,^^2^ The chronic inflammation around the joints leads to substantial damage to the cartilage, bones, tendons, and ligaments, thereby, significantly impairing overall function and quality of life. ^3^ As a result, RA patients have a 50% greater chance of developing cardiometabolic diseases, ^4^ such as hypertension, insulin resistance, obesity, and sarcopenia, thereby increasing the propensity of further disability and mortality. ^1^^,^^5^^–^^7^ Worldwide, RA affects 0.2-1.0% of the total population, with a higher prevalence in women, being 3 to 8 times more frequent than in men. ^8^^,^^9^

Treatments for RA include the prescription of antirheumatic/anti-inflammatory pharmacological and biological medications such as nonsteroidal anti-inflammatory drugs (NSAIDs), corticosteroids, and disease-modifying antirheumatic drugs (DMARDs). They lead to significant improvements in functionality (decreased stiffness) and quality of life; however, they result in increased healthcare costs. Importantly, these treatments have no effect or even augment the cardiometabolic risk factors associated with this disease, probably due to the sedentary lifestyle and poor dietary patterns of RA individuals. ^5^^–^^7^ Furthermore, as these traditional treatments target and inhibit the signaling pathways that produce inflammatory cytokines, including tumor necrosis alpha (TNF-α) and interleukin-6 (IL-6), they lead to immunosuppression and other unwanted side effects. ^10^ Interestingly, there is substantial evidence supporting that consumption or supplementation with omega-3 (n-3) polyunsaturated fatty acids (PUFAs) and regular aerobic exercise (AEx) training have positive effects on RA. For instance, several studies report significant benefits of n-3 PUFA on clinical and functional outcomes in RA individuals, usually when high doses were used (> 2g/d). ^11^^–^^13^ In particular, regular consumption of fish oil rich in n-3 PUFAs leads to the incorporation of eicosapentaenoic acid (C20:5n-3, EPA) and docosahexaenoic acid (C22:6n-3, DHA) into blood and synovial fluid phospholipids. ^14^ This has been shown to improve several disease-specific and functional outcomes of RA, such as morning stiffness, ^15^ joint pain, ^16^ number of swollen and tender joints, ^17^ grip strength, ^18^^,^^19^ decreased NSAID consumption, ^15^^,^^20^ and health-related quality of life. ^13^ In addition, regular consumption of n-3 PUFAs decreases the serum levels of the proinflammatory mediator thromboxane B _2_ (TXB _2_) and the production of prostaglandin E _2_ (PGE _2_) after 24 h incubation of blood with lipopolysaccharide (LPS). ^20^ Moreover, n-3 PUFA supplementation reduces the production of several pro-inflammatory cytokines, including the blood levels of C-reactive protein (CRP), ^21^ TNF-α ^22^ and IL-1β. ^18^ Surprisingly, the specific mechanisms behind the anti-inflammatory and positive health-related effects of n-3 PUFAs in RA individuals are still inconclusive.

The regular practice of physical activity/exercise is also an effective and multifactorial approach for improving several aspects of health in RA subjects, including cardiovascular, metabolic, musculoskeletal, and functional health. ^23^ For instance, RA participants who performed 6 months of regular AEx [3 times per week at 70% of peak oxygen consumption (VO _2_peak)] improved their cardiorespiratory fitness (8% VO _2_peak) and cardiometabolic health. ^24^ Furthermore, AEx training improved symptoms and specific clinical measures of RA, including the perception of fatigue, joint pain, physical function (decreased walk time and increased grip strength), quality of life [health assessment questionnaire HAQ)], and RA disease activity score (23%). ^6^^,^^23^^–^^26^ These beneficial effects are most likely mediated by the anti-inflammatory effects of exercise; however, the mechanisms behind this effect are not yet known.

Because some of the putative mechanisms behind the effects of n-3 PUFA supplementation and AEx training overlap, ^27^^–^^29^ the combination of these treatments in individuals with RA may further improve their health. However, to date, no study has examined the effects of n-3 PUFA supplementation and AEx training in individuals with RA. Further, the emergence of a novel class of lipid metabolites derived from n-3 PUFAs – specialized pro-resolving mediators (SPMs) – which activate pathways that reduce excessive inflammation, yet also enhance pathways necessary for tissue repair and regeneration, ^30^^,^^31^ has initiated a promising new era of research that could explain the benefits observed after n-3 PUFA supplementation and AEx in individuals with RA. However, whether they are responsible for this adaptive response, also remains to be elucidated.

*Research hypothesis:* N-3 PUFA and AEx will have synergistic effects on disease progression, cardiometabolic health, and quality of life in individuals with RA, and these changes will be associated with SPM production in blood and synovial fluid.

*General aim:* To investigate the effects of n-3 PUFA supplementation and AEx training on disease progression, cardiometabolic health, and quality of life and their association with the plasma and synovial fluid levels of SPMs in individuals with RA.

*Specific aims*:

(1) To compare the effects of n-3 PUFA supplementation, AEx training, and the combination of both on disease progression, cardiometabolic health, and quality of life in individuals with RA.
(2) To compare the effects of n-3 PUFA supplementation, AEx, and the combination of both on the plasma and synovial fluid levels of the SPMs in individuals with RA.
(3) To examine the relationship between the plasma levels of the n-3 PUFA derived SPMs with systemic disease progression, cardiometabolic health, and quality of life in individuals with RA

To achieve these aims we have designed a randomized, 16-week, placebo-controlled, blinded interventional trial.

## Protocol

### Study settings

Participants will be recruited from hospitals and private clinics in the central region of Chile, whereas all the interventions regarding AEx will be carried out at the Universidad de O’Higgins and the Hospital Regional de Rancagua. All institutions are in the Región del Libertador Bernardo O’Higgins, Chile.

### Sample size

The sample size calculation for this study is based on an α level of 0.05, a power (1-β) of 0.8, and effect size of 0.42 (morning stiffness). ^11^ A minimum of 18 participants are necessary per group; however, 22 individuals will be recruited to account for a typical dropout rate of 20% in long-term intervention studies.

### Participants

88 individuals (18-65 y) with rheumatoid arthritis (RA) will be recruited via newspaper advertisements, posters, flyers, visits to local community centers, and referrals from associated centers and Rheumatologists. All participants will obtain medical clearance from their Rheumatologist before taking part in the study. Individuals with moderate disease activity, as defined by the Disease Activity Score 28 (DAS28) > 2.6 and < 5.1 will be included. Participants taking nonsteroidal anti-inflammatory drugs (NSAIDs), glucocorticoids, or disease-modifying anti-rheumatic drugs (DMARDs) will be eligible; however, the dosages of these agents must be constant at least four weeks before, must remain within this limit throughout the study, and prednisone dose should not be higher than 7.5 mg/d. ^32^ Individuals diagnosed with gastrointestinal or metabolic diseases, regular alcohol abuse, smokers, and dietary supplement intake (e.g., fish oil capsules) or consumption of fish > 2 times per week will be excluded. Further, individuals that perform regular aerobic exercise (> 150 min moderate intensity per week) or have any physical or biomechanical limitations to complete the exercise program will also be excluded. Finally, subjects will be excluded if they present blood levels of aspartate aminotransferase, alanine transaminase, or creatinine higher than 1.5 times the maximum normal limit and total bilirubin levels of more than 1.8 mg/dL. ^33^^,^^34^ Participants will be encouraged to continue with their everyday normal activities. They will be fully informed of the nature and possible risks of the experimental procedures before providing their written informed consent. Participation in this study is voluntary.

### Study design

This study consists of a 16-week intervention with n-3 PUFAs and/or aerobic exercise (AEx) training. The subjects will be randomly assigned in a double-blinded manner to one of four groups: placebo control (PLA), PLA+AEx, n-3, or n-3+AEx. A stratified randomized assignment (by block) process will be employed to ensure that the experimental groups are balanced for disease activity, pharmacological treatment, sex, and age. The principal investigator and pharmacy personal will be aware of patient allocation and will code the information. Patients and exercise intervention supervisors will be blinded for supplement intervention, while medical specialists and database analysis personal will be blinded for supplement treatment and exercise intervention. The study will be unblinded for patients and medical specialist if the participant worsens their condition according to the criteria described below or if they decide to drop out the study.

The training sessions will be conducted at the Laboratorio de Ciencias del Ejercicio en el Ciclo Vital (Lab-CERVITAL), Universidad de O’Higgins. The week before intervention the participants will perform RA specific tests (e.g., Disease Activity Score-28) and functional measurements (e.g., handgrip strength), peak aerobic capacity test, a dietary and physical activity assessment, and blood samples will be collected. Subsequently, participants will start their 16-week intervention (PLA, PLA+AEx, n-3, or n-3+AEx). At the end of the intervention the same initial measurements, questionnaires, and assessments will be collected. In a subgroup of participants (n=24 in total or n=6 per group) extraction of synovial fluid will be performed before and after the intervention.

### Primary and secondary outcomes

Primary outcome: Early morning stiffness is one of the main outcomes significantly improved by n-3 PUFA supplementation in RA patients. ^11^ Therefore, the difference in the duration (minutes) of early morning stiffness has been selected as the primary outcome to compare the intervention effect in each group after the intervention period.

Secondary outcomes: Other relevant outcomes which allow the assessment of disease progression will be measured, such as the Disease Activity Score-28, DHA-SPMs, tender and swollen joint count, quality of life, grip strength, analgesic use, and TNF-α blood levels.

### Supplementation and exercise intervention

The participants will be supplemented with either 5 capsules per day of Omega UP (Newscience, Chile) equivalent to 2.5 g/d of DHA and 0.5 g/d of EPA or a placebo filled gelatin capsule. The current doses are within the limits recommended by the European Food Safety Authority and have been shown to be safe, to be incorporated into cell membranes, and to produce significant improvements in overall health in individuals with RA. ^35^^–^^38^ Capsules will be placed into de-identified bottles by people not involved in the study and provided to the participants to ensure double blinding.

Patients will be given 2-4 weeks of capsules at a time, with all capsules counted before and after this timeframe, and a small notepad will be provided for them to write down every time when the capsules are taken. Furthermore, patients will receive weekly phone calls and text messages to serve as reminders for treatment adherence. Capsule treatment will be discontinued if patients worsen their condition or if the patient decides to drop-out without questioning their decision.

The exercise intervention will be performed according to the recommendations from the European Alliance of Associations for Rheumatology (EULAR) consisting of aerobic type exercise training three times per week, on non-consecutive days, with a total time of 20-60 minutes. ^25^ The AEx will be performed on a stationary electronically braked cycle ergometer starting at 40-50% of VO _2_peak for 20 minutes. The intensity and volume of cycling will then be gradually increased to target at least 60-70% of VO _2_peak for 50-60 minutes over the final 10 weeks. Heart rate, power output and rating of perceived exertion (RPE) will be monitored during training intervention.

A brief talk about the exercise importance for their conditions will be made every first session of the week to create awareness about it. Also, sessions have been planned at moderate intensity to decrease the risk of discomfort and to improve adherence. The exercise intervention will be stopped if the patient presents any discomfort related to fatigue, consciousness, cardiac, respiratory, or muscular hassle. Furthermore, if at some point disease symptoms are an obstacle for exercise practice, the patient will be advised to drop out the study. Finally, all patients in the exercise group may drop out from the study at any point without justification.

In case of any question or inquiry during the follow-up, the patients will be given a phone number and email to contact study the personnel. Furthermore, during the phone calls made by the personnel a question regarding compliance with the study will be asked. If these measures are not enough for promoting participant retention and the patient decides to drop out, a question regarding their reason for discontinuation will be asked. This information will be analyzed to avoid future study dropouts.

### Monitoring

A data monitoring committee will not be needed in this study since it has minimal risks and will be a short period of intervention. Interim analyses will be made by the principal investigator and a selected team of personnel every two weeks, indicating the total number of reported adverse events and its severity, any other undesirable effect, and the number of dropouts.

### Characterization and anthropometric measures of participants

Body mass and height will be determined with a scale and wall-mounted stadiometer. The participant’s age, body mass index (BMI), educational status, socioeconomic level, years of diagnosis, smoking habits, comorbidities, and medications will be recorded.

### RA disease progression

*Duration of morning stiffness*: This will be assessed by asking “How long is your morning stiffness from waking until maximum improvement?”. ^39^ The duration of morning stiffness will be reported in minutes up to a maximum of 180 minutes.

*Disease Activity Score-28 (DAS28*): Describes the severity of rheumatoid arthritis using clinical and laboratory data. It is composed of the number of swollen joints (total 28), the number of tender joints (total 28), the Westgren erythrocyte sedimentation rate (ESR), and patients’ global disease assessment through a visual analog scale (VAS). The DAS28 has been extensively validated for its use in clinical trials and it is required by the Chilean Ministry of Health. ^40^

*Health assessment questionnaire disability index (HAQDI):* This tool evaluates the functional status of RA individuals. It consists of eight sections: dressing, arising, eating, walking, hygiene, reach, grip, and activities, with 2 or 3 questions for each section. Scoring is from 0 (without any difficulty) to 3 (unable to do). The 8 scores of the 8 sections are summed and divided by 8. This questionnaire has been validated to be used among Chilean RA patients. ^41^

*Quality of life RA (QOL-RA) scale*: This scale is an RA-specific health-related quality of life instrument consisting of an 8-item scale. Each item starts with the definition of an element to be considered in rating one’s quality of life, followed by a horizontal 10-point scale anchored with 1 (very poor) at one end and 10 (excellent) at the other end. The elements are physical ability, pain, interaction with family and friends, support from family and friends, mood, tension, arthritis, and health. The higher the QOL-RA Scale score, the higher the health-related quality of life. This scale has been validated to be used in the Spanish language. ^42^

*Analgesic use*: Analgesic use will be measured using pill counts for paracetamol and NSAIDs.

### Functional measurements

*Timed Up and Go test*: The participant starts in a seated position, stands up upon command, walks 3 meters, turns around, walks back to the chair, and sits down. The time stops when the subject is seated.

*Short Physical Performance Battery (SPPB*): This battery of tests evaluates lower extremity functional performance using timed measures of standing balance, a 4-meter walk, and five repetitive chair stands. ^43^

*Handgrip strength*: Measures the maximum isometric strength of the hand and forearm muscles. The participant holds the dynamometer in the hand with the arm at a right angle and the elbow by the side of the body. When ready, the participant squeezes the dynamometer with maximum isometric effort for 3-5 seconds. Participants will be instructed to perform the test as fast and hard as possible and verbal encouragement will be provided simultaneously. The highest value out of three contractions will be used for analysis.

### Peak aerobic capacity

The VO _2_ peak will be determined during an incremental cycling test to exhaustion using a recumbent cycle ergometer following the recommendations of the American College of Sports Medicine, ^44^ using a portable and validated gas analyzer. ^45^ Blood pressure and heart rate will also be analyzed at rest and during the exercise test.

### Dietary and physical activity assessment

The participants will have to fill a dietary record using image-assisted method over three days (two consecutive weekdays and one weekend day) before and after the supplementation period for estimation of macronutrient intake. ^46^ Similarly, participants will answer the Global Physical Activity Questionnaire (GPAQ) to assess their physical activity. ^47^

### Blood samples

Blood samples will be collected after an overnight fast (12 h) using a needle and Vacutainer kit and will be immediately centrifuged to obtain plasma (1000 × g for 10 min at 4°C) and erythrocyte fractions (3000 × g for 10 min at 20°C). Both fractions will be frozen at −80°C until further analysis. A portion of venous blood will also be collected in LeukoLOCK™ Total RNA Isolation System for the capture and purification of intact RNA from white blood cells, which will be frozen and stored at –80°C until further processing.

### Clinical, cardiometabolic, and inflammatory blood measurements

Plasma rheumatoid factor (RF) and lipid profile will be determined. Further, plasma interleukin-10, and TNF-α levels will be measured via commercial ELISA assay kits.

### Leukocyte gene expression

cDNA will be prepared by reverse transcription of 1 ug of total RNA, using the SensiFASTTM cDNA Synthesis Kit (Bioline) according to manufacturer’s protocol. RNA quantity and quality will be assessed using a Nanodrop 8000 (ThermoScientific, Wilmington, DE, USA) and an Agilent 2100 BioAnalyzer (Agilent Technologies Inc., Santa Clara, California, USA), respectively. Real-time RT-qPCR will be carried out to quantify the changes in the expression of genes associated with inflammatory pathways and SPM synthesis, including TNF-α, interleukin-1β (IL-1β), IL-6, cyclooxygenase-2 (COX-2), lipoxygenase-5 (ALOX5), lipooxygenase-12 (ALOX12), lipooxygenase-15 (ALOX15), and calcium-independent phospholipase A2 VIA (iPLA2VIA). 18S ribosomal RNA (18SRNA) and glyceraldehyde 3-phosphate dehydrogenase (GAPDH) will be used as housekeeping genes. Data will be analyzed using the ΔΔCt method.

### Synovial fluid extraction

All individuals must obtain medical clearance from their Rheumatologist. RA subjects will attend the laboratory after an overnight fast, without having carried out any type of exercise in the previous 48 hours. Local anesthesia (1% Lidocaine) will be applied to the affected joint and synovial fluid will be obtained by a certified rheumatologist. ^48^ Synovial fluid will be immediately placed on ice and aliquoted for specific analyses. All aliquots will be stored at −80°C until use.

### Phospholipid fatty acid composition

Quantitative extraction of total lipids from erythrocytes, plasma, and synovial fluid will be carried out according to Bligh and Dyer ^49^ with the addition of BHT. Fatty acid methyl esters (FAMEs) from erythrocytes and plasma will be prepared according to Morrison and Smith. ^50^ FAMEs will be extracted with 0.5 mL of hexane. FAMEs will be identified and quantified using a gas-liquid chromatograph (Agilent7890B, Santa Clara, CA, USA) equipped with a capillary column (Agilent HP-88, 100 m × 0.250 mm; I.D. 0.25 μm) and flame ionization detector (FID). Identification and quantification of FAMEs will be achieved by comparing the retention times and the peak area% values of unknown samples to those of a commercial lipid standard (Nu-Chek Prep Inc., Elysian, MN, USA). C23:0 will be used as internal standard (Nu-Chek Prep Inc., Elysian, MN, USA) and data will be processed using the Hewlett-Packard Chemstation software system.

### Analyses of SPMs

Plasma and synovial fluid samples (400 μL) will be analyzed in duplicate for oxylipins by HPLC/MS/MS, as previously described. ^51^ Briefly, deuterated internal standards (Cayman Chemicals) will be added to plasma samples and adjusted to pH <3.0 prior to solid-phase extraction using Strata-X-SPE (Phenomenex) columns preconditioned with methanol, followed by pH 3.0 water. Samples will be loaded on the columns, washed, and free SPMs will be eluted with 100% methanol. The eluate will subsequently be dried and resuspended in solvent A (water/acetonitrile/formic acid, 70/30/0.02 v/v/v) for oxylipin analysis by HPLC/MS/MS, using a Luna 5-μm C18 column (100 Å, 250 × 2.0 mm, Phenomenex) on a Shimadzu Nexera XR HPLC, coupled to an ABSciex QTRAP 6500 MS operating in negative mode with electrospray ionization. Oxylipins will be eluted with the following gradient: 100% solvent A between 0 and 1 min, and then solvent B (acetonitrile/isopropyl alcohol, 50/50, v/v) is increased linearly to 25% at 3 min, to 45% at 11 min, to 60% at 13 min, to 75% at 18 min, and to 90% at 18.5 min. Solvent B will then be held at 90% until minute 20, dropped to 0% by 21 min, and held until 25 min. Quantification of oxylipins will be performed using a stable isotope dilution method.

### Statistical analysis

Results will be expressed as mean ± SD. A two-way ANOVA with repeated measures followed by Fisher’s least significant difference post-test for multiple comparisons between groups will be used. Linear regression models will be constructed to examine the association between SPMs and clinical, functional, and health parameters, accounting for participant age, sex, and BMI as covariates. A value of p<0.05 will be considered statistically significant.

**Dissemination**

The results will be communicated to the funding body through a formal report. Further, they will be communicated to the participants and the interested public at the Universidad de O’Higgins and will be disseminated through presentations at national and international scientific conferences. The study outcomes will be published through peer-reviewed journals, no matter the trial outcome. There is no limit to the publication of the trial results.

**Study status**

The recruitment of participants will begin during the winter (June-September) of 2023. The data collection of this study will be completed in December 2024.

## Discussion

RA is a chronic autoimmune and inflammatory disease characterized by inflammation around the joints leading to substantial damage of the cartilages, bones, tendons, and ligaments, significantly affecting overall function, quality of life, and increasing the propensity of mortality. The current treatments for patients with RA (DMARDs, NSAIDs, and anti-TNF biologics) intend to disrupt the inflammatory response and slow disease progression; however, their chronic consumption may lead to unwanted side effects (immunosuppression and infections) and prevent optimal resolution and tissue repair, which is imperative in RA. Further, the lack of robust and sensitive biomarkers to determine treatment responsiveness in RA has impeded proper tracing and development of novel individualized interventions. Therefore, elucidating the SPMs, as well as the underlying inflammatory and SPM synthesis transcriptional pathways, associated with the improvements in disease progression, cardiometabolic health, and quality of life after n-3 PUFA supplementation and AEx training could lead to the development of new markers and individualized treatment strategies that improve overall health and quality of life in RA individuals. Finally, these results could prompt the development of new pharmacological treatments that enhance the production of the SPMs observed after n-3 PUFA supplementation and AEx training to improve treatment outcomes in RA individuals.

### Ethical considerations

Due to ethical considerations (divergent inflamed joints between participants), we will only perform the synovial fluid procedure in a small subset of patients from each intervention group. We will ensure that all individuals receive the best medical attention before, during, and after the procedure. Before a participant is eligible, a full medical screening will be performed to assess any risks. The individuals will be followed up routinely 48 h after the procedure to check if any complication occurs, which is unlikely. If a complication occurs, the principal investigator of this study will take care of all associated medical procedure costs and will seek the best treatment for the participant.

This study received ethics approval from the Comité de Ética de Investigación en Seres Humanos #219-2021 (Universidad de Chile, Santiago, Chile). All participants must sign a consent form prior participation in the study.

### Registration and reporting guidelines

This trial has been registered with ClinicalTrials.gov, #NCT05945693. The construction of this study protocol followed the Interventional Trials 2013 (SPIRIT) guidelines.

## Data Availability

No data are associated with this article.
