# Supplementary material for: Role of specialized pro-resolving mediators on inflammation, cardiometabolic health, disease progression, and quality of life after omega-3 PUFA supplementation and aerobic exercise training in individuals with rheumatoid arthritis: a randomized 16-week, placebo-controlled interventional trial *
Source: F1000Res. 2023 Aug 7;12:942. [Version 1] doi: 10.12688/f1000research.138392.1 (PMC11109545; doi:10.12688/f1000research.138392.1)
Supplement: Peer review history

# Reviewer report: Reviewer response for version 1

**DOI:** 10.5256/f1000research.151582.r276499

**Published:** 2024-06-08

**Authors:** Chris Mcglory, Danielle Nyman

**Affiliations:** 1 School of Kinesiology and Health Studies, Queen's University, Kingston, Ontario, Canada; 2 Queen's University (Ringgold ID: 4257), Kingston, Ontario, Canada

Thank you for the opportunity to review this interesting manuscript. After careful review we have highlighted some points/comments that we believe will improve the overall quality of the study and reporting of the final manuscript.

**Introduction:**

1. Are there specific/published measures of function for RA? What are the key outcomes used to support this point?
2. More info would benefit the connection between RA and development of metabolic disease.
3. What is the burden of increased healthcare cost- is it on the individual or society?
4. Specify meaningful clinical and functional outcomes
5. Is the n-3 PUFA incorporated freely into the blood, or is it its incorporation into blood cells that have possible positive effects?
6. Expand on the proposed function/mechanisms SPMs act through.
7. Is there proposed interaction between the AEx and N-3 only groups? How might these compare to the combined group? Do you suspect and additive effect of the combined intervention?

**General methods:**

1. What is the time frame of intervention vs. observation? Is this suitable to measure disease progression?
2. Sample size: Why use morning stiffness for power calculation? Will this be the primary outcome? Is this the most influential factor clinically?
3. What is the significance of this prednisone dose and why is it grounds for exclusion?
4. Validation for excluding drinkers and smokers?

**General design:**

1. Is there a specific time frame to recruit 88 participants? If there is more than 22% drop out, will you recruit more than 22 people per group?
2. Can this study be considered double blind if the PI is aware of group allocation?
3. What is the peak aerobic test used? What does the physical activity assessment consist of- is it self-reported?
4. Would it be worthwhile to include more timepoints? Even just for blood and synovial fluid to see the time course of n-3 saturation?
5. Why not try to recruit everyone for synovial fluid extraction?
6. Is morning stiffness clinically meaningful? Does this measure alone influence quality of life or similar outcomes
7. Why were these doses of DHA and EPA chosen specifically?
8. Is there any concern that gelatin may confound results due to possible implications on joint health?
9. Will the progression of exercise be standardized relative to time?
10. Are the phone calls with compliance questions the same call/frequency as the adherence reminder?
11. When is the participant asked to report this measure? On the phone, in person, or in writing? Will this involve active recall? Could this influence their reporting?
12. Again, what is the clinical significance of morning stiffness?
13. Will analgesic used be reported with respect to body mass or sex?
14. Handgrip assessment should be across a standardized time

**Discussion:**

1. What are some of the primary markers of inflammation for this disease?

**Other (pre reported RCT):**

1. Status is listed as not recruiting, but is supposed to be done by Dec 2024?
2. Omega-3 combined with exercise is not listed as a separate intervention
3. Can masking be considered quadruple if the PI knows group allocation

Chris McGlory and Danielle Nyman.

Is the study design appropriate for the research question?

Yes

Is the rationale for, and objectives of, the study clearly described?

Yes

Are sufficient details of the methods provided to allow replication by others?

Yes

Are the datasets clearly presented in a useable and accessible format?

Not applicable

Reviewer Expertise:

Skeletal muscle, nutrition

We confirm that we have read this submission and believe that we have an appropriate level of expertise to confirm that it is of an acceptable scientific standard, however we have significant reservations, as outlined above.

# Reviewer report: Reviewer response for version 1

**DOI:** 10.5256/f1000research.151582.r276501

**Published:** 2024-05-21

**Authors:** Philip C Calder

**Affiliations:** 1 School of Human Development and Health, Faculty of Medicine, University of Southampton, Southampton, England, UK

This manuscript describes a study protocol which has already been peer reviewed and which already has ethical approval. It is well written and clear, the study design is good (2 x 2 factorial design), dosing level of omega-3 is good, combination of omega-3 and exercise a novelty, and clinical and lab outcomes are all relevant. I did not quite understand the rationale for why only a "small subset of patients" will be subject to synovial fluid isolation and it is unclear what "a small subset" means and how that might adversely impact the value of the data obtained.

Is the study design appropriate for the research question?

Yes

Is the rationale for, and objectives of, the study clearly described?

Yes

Are sufficient details of the methods provided to allow replication by others?

Yes

Are the datasets clearly presented in a useable and accessible format?

Not applicable

Reviewer Expertise:

Omega-3 fatty acids; Inflammation; Lipid mediators; Clinical trials

I confirm that I have read this submission and believe that I have an appropriate level of expertise to confirm that it is of an acceptable scientific standard.

# Reviewer report: Reviewer response for version 1

**DOI:** 10.5256/f1000research.151582.r231475

**Published:** 2024-02-14

**Authors:** Łukasz Poniatowski

**Affiliations:** 1 Central Clinical Hospital of the Ministry of the Interior and Administration, Warsaw, Poland

The original article by Jannas-Vela et al "Role of specialized pro-resolving mediators on inflammation, cardiometabolic health, disease progression, and quality of life after omega-3 PUFA supplementation and aerobic exercise training in individuals with rheumatoid arthritis: a randomized 16-week, placebo-controlled interventional trial" covers a potentially interesting and emerging topic related to the rheumatoid arthritis. I regard the main point of this paper as highly attractive as well as the results are clearly presented. The text does not contain any major errors, therefore I have some minor comments and recommendations:

 1. There is a need to provide slightly more expanded introduction shortly mentioning/describing pathogenesis of RA and its impact of modern healthcare.

 2. The figure summarizing and clarifying the results should be added.

 3. Following references should be added and properly cited within the main text:

 - Turczyn P, Wojdasiewicz P, Poniatowski ŁA, Purrahman D, Maślińska M, Żurek G, Romanowska-Próchnicka K, Żuk B, Kwiatkowska B, Piechowski-Jóźwiak B, Szukiewicz D. Omega-3 fatty acids in the treatment of spinal cord injury: untapped potential for therapeutic intervention? Mol Biol Rep. 2022 Nov;49(11):10797-10809. doi: 1

 0.1007/s11033-022-07762-x.

 - Wojdasiewicz P, Poniatowski ŁA, Turczyn P, Frasuńska J, Paradowska-Gorycka A, Tarnacka B. Significance of Omega-3 Fatty Acids in the Prophylaxis and Treatment after Spinal Cord Injury in Rodent Models. Mediators Inflamm. 2020 Jul 29;2020:3164260. doi: 10.1155/2020/3164260. PMID: 32801994; PMCID: PMC7411484.

 4. In some places the use of English could be improved on.

 Completing this gaps will have an impact on the understanding the aim of the study and, from my point of view, is absolutely necessary.

Is the study design appropriate for the research question?

Yes

Is the rationale for, and objectives of, the study clearly described?

Yes

Are sufficient details of the methods provided to allow replication by others?

Yes

Are the datasets clearly presented in a useable and accessible format?

Yes

Reviewer Expertise:

Orthopaedics

I confirm that I have read this submission and believe that I have an appropriate level of expertise to confirm that it is of an acceptable scientific standard.
